# Cognitive insight and quality of life among psychiatric outpatients

**DOI:** 10.1186/s12888-019-2163-y

**Published:** 2019-06-28

**Authors:** Vathsala Sagayadevan, Anitha Jeyagurunathan, Ying Wen Lau, Saleha Shafie, Sherilyn Chang, Hui Lin Ong, Ellaisha Samari, Swapna Kamal Verma, Siow Ann Chong, Mythily Subramaniam

**Affiliations:** 1 Research Division, Institute of Mental Health, Buangkok Green Medical Park, 10, Buangkok View, Singapore, 539747 Singapore; 2 Early Psychosis Intervention Programme (EPIP) & General Psychiatry (GP1), Institute of Mental Health, Buangkok Green Medical Park, 10 Buangkok View, Singapore, 539747 Singapore

**Keywords:** Outpatients, Quality of life, Mental disorders, Insight, Awareness

## Abstract

**Background:**

Past studies have focused primarily on clinical insight and less on cognitive insight among individuals with mental illness.

**Methods:**

This study examined the level of cognitive insight (CI) and its association with quality of life (QoL) among psychiatric outpatients (*N* = 400) in Singapore. The Beck Cognitive Insight Scale (BCIS) consisting of two subscales (self-reflectiveness (SR) and self-certainty (SC)) was used to measure CI while the brief version of the World Health Organization Quality of Life (WHOQOL-BREF) questionnaire was used to assess the subjective well-being of the individual.

**Results:**

Socio-demographic correlates of CI, differences in SR, SC, and CI scores across diagnostic groups, and the association between insight and QoL were examined. Significant differences across diagnostic groups were found only for SR scores. Higher SR and overall CI scores were significantly associated with higher QoL in the environmental domain whereas higher SC scores were associated with lower QoL in the social relationships domain.

**Conclusions:**

An understanding of cognitive insight is necessary to produce a significant change in the underlying belief system of an individual. Together with clinical insight, these two forms of insight can be used to inform therapeutic approaches to increase awareness and improve the QoL of those with mental illnesses.

## Background

Understanding the level of insight of individuals is crucial given that persistent unawareness of an illness and its consequences can be detrimental to the recovery process and prognosis of an individual [1]. Past studies have primarily focused on the concept of clinical insight, which can be defined as an individual’s awareness of psychopathological symptoms, the consequences of the disorder and the need to seek treatment [1–3]. Recent studies however, have attempted to extend this concept by examining cognitive insight (CI) [4], which refers to the awareness of cognitive deficiencies such as impairment of objectivity, resistance to corrective feedback, and overconfidence in decisions [4, 5]. Research on CI has clinical importance given its implication in the formation and maintenance of hallucinations and delusions [4–7] as well as its role in gains achieved during psychotherapy [8, 9].

Studies examining CI have predominantly focused on those with schizophrenia and related psychosis [5, 10] and less so in other populations such as among patients with bipolar disorder, and major depressive disorder (MDD) [11]. In general, individuals with psychosis tend to exhibit poorer insight as compared to those with other illnesses, given their attenuated capacity to reflect on their experiences and correct their distortions [4, 5, 9, 12]. Beck et al. [4] for instance, found CI among inpatients with a psychotic diagnosis (i.e., Schizophrenia, Schizoaffective, MDD with psychotic features) to be lower than that of inpatients without a psychotic diagnosis (i.e., MDD without psychotic features). Likewise, Kao et al. [13] found a Taiwanese sample of patients with psychosis to have significantly lower CI (i.e., less reflective, more confidence in their beliefs) compared to the non-psychotic control groups [13].

Similar to clinical insight [1–3, 14], CI has been associated with an individual’s quality of life (QoL) and functioning [1, 3, 6, 15, 16]. Kim et al. [10] for instance, found higher levels of CI to be associated with lower subjective QoL. Likewise, Xiang et al. [3] found poorer CI scores in patients with schizophrenia to be associated with higher scores on the physical and mental components of QoL. Other studies have found the association with QoL to differ depending on the subscale of CI; while Phalen et al. [6] found greater SR to be associated with better QoL in general, SC was found to be associated with better QoL only among those with more severe symptoms. Rathee et al. [16] on the other hand found CI scores and SR scores among individuals with ongoing auditory hallucinations to be positively correlated with objectively rated QoL scores but not SC scores.

Existing studies on insight have focused on patients with schizophrenia and related psychosis and less on other mental illnesses; a large proportion of these studies have also been conducted in Western countries, [5]. Furthermore, the role of socio-demographic factors in CI has been ambiguous, [13]. The current study thus, aimed to address these gaps in the literature by examining the levels of CI and its association with QoL in a sample of psychiatric outpatients with a clinical diagnosis of schizophrenia and related psychoses, any mood or anxiety disorder in Singapore.

## Methods

### Sample

Singapore is a multiethnic country in Southeast Asia with a population of 5.6 million; Chinese encompassed 74.3% of the resident population, followed by Malays (13.4%), Indians (9.0%), and other ethnic groups (3.2%) [17].

The current study utilized data from a larger cross-sectional study examining the pathways to care and illness perception among psychiatric outpatients (*N* = 400) seeking treatment at the Institute of Mental Health (IMH) or its satellite clinics between October 2015 and December 2016 (Refer to Jeyagurunathan et al. [18] for details on the main study). Participants were included in the study if they were between 21 and 65 years old, capable of providing written consent, able to read and understand English, Chinese, Malay or Tamil, had a clinical diagnosis of schizophrenia and related psychosis, any mood disorder (depression or bipolar disorder), or any anxiety disorder (generalized anxiety disorder, obsessive compulsive disorder, post-traumatic stress disorder or panic disorder) and had an illness duration of not more than 2 years as determined by a psychiatrist using International Classification of Diseases 9th Revision (ICD-9R) criteria. Following written informed consent, participants completed a set of questionnaires, after which they were reimbursed for their time. Individuals who had difficulty concentrating or reading had a research assistant read out the questions to them and clarify their queries. The study received ethics approval from the National Healthcare Group Domain Specific Review Board.

### Measures

#### Socio-demographic questionnaire

Details on age, gender, ethnicity, marital status, education level, and employment status were obtained. Primary diagnosis was also obtained from patients through self-report which was then verified against their medical records.

#### The Beck Cognitive Insight Scale (BCIS)

The BCIS was utilized to understand patients’ perspectives about their anomalous experiences, and their interpretations of specific life events [4]. The 15-item scale encompasses two subscales: self-reflectiveness (SR; 9 items) and self-certainty (SC; 6 items). SR measures patients’ capacity and willingness to observe their mental processes, ability to consider alternative explanations, and openness to feedback whereas SC measures an individual’s overconfidence in their beliefs and resistance to correction [4]. Statements were rated on a scale of 0 (do not agree at all) to 3 (agree completely), with higher SC scores reflective of poorer insight and higher SR scores indicative of better insight [5]. An overall score of CI is derived by subtracting the SC score from the SR score; a higher score would indicate greater CI [3, 4]. The BCIS has demonstrated adequate convergent and construct validity and is able to distinguish patients with psychosis from healthy controls and patients without psychosis [4, 9, 19]. The internal consistency of BCIS in the current sample was 0.76.

#### World Health Organization Quality of Life – Brief Version (WHOQOL-BREF)

The WHOQOL-BREF questionnaire assessed the subjective well-being of an individual with reference to the past 2 weeks. The 26-item questionnaire examines the multi-dimensional concept of QoL through the physical health, psychological, social relationships, and environmental aspects of well-being [20]. Items are scored on a Likert scale of 1 to 5, enquiring on “how much”, “how completely”, “how often”, “how good” or “how satisfied” the individual felt with reference to each item; higher scores would indicate a better QoL [20, 21]. This tool has been cross-culturally validated and is available in more than 40 languages [22].

### Statistical analysis

Analysis was performed using the Statistical Package for Social Sciences (SPSS) version 23. Descriptive statistics was used to obtain the frequency distribution of the study sample. One-way ANOVA was used to examine the differences in SR, SC, and CI scores across diagnostic groups (schizophrenia and related psychosis, any mood or anxiety disorders). Multiple linear regressions were conducted to examine the socio-demographic correlates of CI as well as the association between CI and QoL. All statistically significant results were reported at *p* < .05.

## Results

### Sample characteristics

Table 1 shows the socio-demographic distribution of the sample. Of the total sample (*N* = 402), data from 400 patients were used for analysis; two were withdrawn due to the total duration of mental illness being more than 2 years. Of the 400 outpatients, 30% had anxiety disorders (*n* = 120), 35% had mood disorders (*n* = 140), and 35% had schizophrenia and related psychosis (*n* = 140). Majority of the sample was male (52.8%), of Chinese ethnicity (62.8%), had ‘A’ level/Poly/Other Diploma (i.e., qualifications obtained at a post-secondary/pre-university level) (39.0%), were employed (58.3%), and single (64.8%). The mean age of the sample was 32.66 years (SD = 11.38). The mean BCIS score obtained in this sample was 5.61 (range:-10 to 23).

**Table 1:** Socio-demographic Characteristics of Sample

Variable		Sample (*N* = 400)		Schizophrenia and related psychosis (*n* = 140)		Any Mood Disorder (*n* = 140)		Any Anxiety Disorder (*n* = 120)	
		n	%	n	%	n	%	n	%
Age group	21–34	266	66.5	93	66.4	98	70.0	75	62.5
	35–49	85	21.3	38	27.1	26	18.6	21	17.5
	50–65	49	12.3	9	6.4	16	11.4	24	20.0
Gender	Female	189	47.3	68	48.6	72	51.4	49	40.8
	Male	211	52.8	72	51.4	68	48.6	71	59.2
Ethnicity	Malay	90	22.5	34	24.3	35	25.0	21	17.5
	Indian	59	14.8	17	12.1	28	20.0	14	11.7
	Chinese	251	62.8	89	63.6	77	55.0	85	70.8
Education Level	No formal education/PSLE	26	6.5	8	5.7	10	7.1	8	6.7
	Secondary/O level/N level/ Vocational	141	35.3	57	40.7	50	35.7	34	28.3
	Tertiary and above	77	19.3	30	21.4	22	15.7	25	20.8
	A level/Poly/Other Diploma	156	39.0	45	32.1	58	41.4	53	44.2
Marital Status	Married	107	26.8	30	21.4	44	31.4	33	27.5
	Separated	34	8.5	11	7.9	16	11.4	7	5.8
	Single	259	64.8	99	70.7	80	57.1	80	66.7
Employment Status	Unemployed	167	41.8	80	57.1	50	35.7	37	30.8
	Employed	233	58.3	60	42.9	90	64.3	83	69.2

A Level: Singapore-Cambridge General Certificate of Education Advanced Level

N Level: Singapore-Cambridge General Certificate of Education Normal Level

O Level: Singapore-Cambridge General Certificate of Education Ordinary Level

Poly: Polytechnic

PSLE: Primary School Leaving Examination

### SR, SC, and CI scores across diagnostic groups

A one-way ANOVA was conducted to examine CI and its subscales across diagnostic groups (Table 2). There was a statistically significant difference in SR scores across the diagnostic groups; *F* (2, 397) = 3.96, *p* = .020. Post-hoc comparisons indicated individuals with schizophrenia and related psychosis (M = 11.80, SD = 5.04) to have significantly lower SR mean scores than those with anxiety disorders (M = 13.93, SD = 8.56). No significant differences were found for SC scores and overall CI scores with respect to diagnosis.

**Table 2:** Means and Standard Deviations of the Beck Cognitive Insight Scale (BCIS) Scores by Diagnostic Groups

		Cognitive Insight (CI)			Self-Reflectiveness (SR)			Self-Certainty (SC)		
		N	M	SD	N	M	SD	N	M	SD
Diagnostic group	Any Mood Disorder	140	5.22	5.10	140	12.51	4.50	140	7.29	3.64
	Any Anxiety Disorder	120	7.12	9.38	120	13.93	8.56	120	6.82	3.85
	Schizophrenia and related Psychosis	140	5.34	5.96	140	11.80	5.04	140	6.46	3.73

### Socio-demographic correlates of CI and subscales

Multiple linear regression analyses were conducted to examine the socio-demographic correlates of CI and its subscales (Table 3). With regards to SR scores, those with anxiety disorders were more likely to have higher SR scores than those with schizophrenia and related psychosis (β = 1.827, *p* = .003); whereas those between the age 50–65 years (β = − 2.288, *p* = .011) were less likely to report higher SR scores compared to those between 21 and 34 years old. Only educational level was significantly associated with SC scores. In particular, those with no formal education/Primary school leaving examination (PSLE) (β = 2.025, *p* = .023) were more likely to report higher SC scores than those with A level/polytechnic diploma. In relation to overall CI scores, those between the age of 50–65 years (β = − 2.368, *p* = .020) were less likely to have higher overall CI scores than those between 21 and 34 years old. In addition, those of Malay ethnicity (β = − 1.412, *p* = .039) tended to report lower CI scores than the Chinese.

**Table 3:** Socio-demographic correlates of Beck Cognitive Insight Scale (BCIS)

Beck Cognitive Insight Scale (BCIS)												
	Cognitive Insight (CI) (Composite)				Self-Reflectiveness (SR)				Self-Certainty (SC)			
	β	95% CI^†^		*p*	β	95% CI^†^		*p*	β	95% CI^†^		*p*
Socio-demographic Variables												
Age												
35–49	−.968	−2.467	.532	.205	−.781	−2.098	.537	.245	.206	−.831	1.242	.697
50–65	−2.368	−4.365	−.371	**.020**	−2.288	−4.042	−.534	**.011**	.109	−1.270	1.489	.876
21–34	Reference											
Gender												
Female	.672	−.451	1.794	.240	.773	−.213	1.759	.124	.117	−.659	.892	.768
Male	Reference											
Ethnicity												
Malay	−1.412	−2.751	−.073	**.039**	−.501	− 1.677	.676	.403	.932	.007	1.857	**.048**
Indian	−1.306	−2.845	.233	.096	−.447	−1.799	.904	.516	.876	−.187	1.939	.106
Chinese	Reference											
Education Level												
No formal education/PSLE	.068	−2.457	2.594	.958	2.078	−.141	4.296	.066	2.025	.280	3.770	**.023**
Secondary/O level/N level/ Vocational	−.466	−1.759	.826	.478	.141	−.994	1.276	.807	.633	−.259	1.526	.164
Tertiary and above	.582	−.918	2.082	.446	−.355	−1.672	.963	.597	−.898	−1.933	.138	.089
A level/Poly/Other Diploma	Reference											
Employment Status												
Unemployed	.140	−.982	1.261	.807	.181	−.804	1.166	.718	.059	−.715	.834	.880
Employed	Reference											
Marital Status												
Married	−.771	−2.251	.708	.306	−1.024	−2.324	.275	.122	−.253	− 1.276	.769	.626
Separated	−.868	−3.017	1.281	.428	−.570	−2.458	1.318	.553	.289	−1.195	1.774	.702
Single	Reference											
Diagnostic Group												
Any Mood Disorder	.155	−1.159	1.468	.817	.897	−.257	2.051	.127	.748	−.159	1.656	.106
Any Anxiety Disorder	1.250	−.125	2.624	.075	1.827	.619	3.034	**.003**	.532	−.416	1.480	.271
Schizophrenia and related Psychosis	Reference											
R Squared Values	0.071				0.058				0.07			

^†^*CI* Confidence interval

*p* values <.05 are reflected in bold

A Level: Singapore-Cambridge General Certificate of Education Advanced Level

N Level: Singapore-Cambridge General Certificate of Education Normal Level

O Level: Singapore-Cambridge General Certificate of Education Ordinary Level

Poly: Polytechnic

PSLE: Primary School Leaving Examination

### Association between CI and QoL

A significant positive correlation was only found between overall CI scores and the environmental domain of QoL (r = .153, *p* < .01). Multiple linear regression analyses after adjusting for socio-demographic variables and diagnostic groups found higher CI scores to be significantly associated with higher QoL in the environmental domain (β = .480, *p* = .003) (Table 4). With regards to the subscales, after controlling for socio-demographic and diagnostic correlates, higher SR scores were associated with higher QoL in the environment domain (β = .404, *p* = .030) whereas, higher SC scores were associated with lower scores on the social relationships domain of QoL (β = −.577, *p* = .045).

**Table 4:** Association between Quality of Life and Cognitive Insight

Quality of Life (QOL)																
	Physical Health				Social Relationships				Psychological				Environment			
	β	95% CI^†^		*p*	β	95% CI^†^		*p*	β	95% CI^†^		*p*	β	95% CI^†^		*p*
Socio-demographic Variables																
Age																
35–49	1.745	−1.729	5.219	.324	.143	−5.700	5.986	.962	1.251	−2.984	5.486	.562	−.198	−4.961	4.565	.935
50–65	6.263	1.615	10.911	**.008**	14.288	6.474	22.100	**.000**	9.887	4.221	15.554	**.001**	12.144	5.772	18.517	**.000**
21–34	Reference															
Gender																
Female	1.182	−1.418	3.781	.372	1.573	−2.816	5.962	.481	.949	−2.220	4.118	.556	.892	−2.672	4.455	.623
Male	Reference															
Ethnicity																
Malay	3.618	.505	6.731	**.023**	4.870	−.366	10.100	.068	6.676	2.881	10.470	**.001**	3.562	−.705	7.830	.102
Indian	2.522	−1.047	6.092	.166	−3.978	−10.023	2.066	.196	4.969	.618	9.321	**.025**	−.310	−5.204	4.584	.901
Chinese	Reference															
Education Level																
No formal education/PSLE	−5.291	−11.128	.547	.076	−8.947	−18.755	.861	.074	−4.578	−11.694	2.539	.207	−9.370	−17.373	−1.366	**.022**
Secondary/O level/N level/ Vocational	−.533	−3.522	2.457	.726	−1.560	−6.610	3.489	.544	−1.243	−4.887	2.401	.503	−4.491	−8.589	−.392	**.032**
Tertiary and above	.494	−2.975	3.964	.780	1.632	−4.214	7.477	.583	.449	−3.781	4.678	.835	5.936	1.179	10.692	**.015**
A level/Poly/Other Diploma	Reference															
Employment Status																
Unemployed	−2.418	−5.011	.175	.068	−2.363	−6.740	2.013	.289	−3.648	−6.809	−.486	**.024**	−4.595	−8.151	−1.040	**.011**
Employed	Reference															
Marital Status																
Married	1.112	−2.312	4.537	.524	1.435	−4.319	7.188	.624	1.370	−2.805	5.544	.519	−.858	−5.553	3.837	.719
Separated	−.332	−5.303	4.639	.896	−9.709	−18.062	−1.360	**.023**	−1.288	−7.349	4.772	.676	−4.350	−11.166	2.465	.210
Single	Reference															
Diagnostic Group																
Any Mood Disorder	−6.810	−9.846	−3.774	**.000**	−8.951	−14.077	−3.825	**.001**	−8.105	−11.807	−4.404	**.000**	−6.802	−10.964	−2.639	**.001**
Any Anxiety Disorder	−4.598	−7.787	−1.408	**.005**	−3.350	−8.735	2.034	.222	−6.281	−10.169	−2.393	**.002**	−4.364	−8.736	.009	**.050**
Schizophrenia and related Psychosis	Reference															
Cognitive Insight	.119	−.112	.351	.311	.373	−.019	.766	.062	.159	−.124	.441	.270	.480	.162	.797	**.003**
R Squared Values	0.086				0.107				0.105				0.133			

^†^*CI* Confidence interval

*p* values <.05 are reflected in bold

A Level: Singapore-Cambridge General Certificate of Education Advanced Level

N Level: Singapore-Cambridge General Certificate of Education Normal Level

O Level: Singapore-Cambridge General Certificate of Education Ordinary Level

Poly: Polytechnic

PSLE: Primary School Leaving Examination

## Discussion

The current study examined cognitive insight among outpatients with a range of psychiatric disorders and its association with QoL. The mean BCIS score obtained in the current study was 5.61. This was similar to the mean BCIS score obtained by Colis et al. [11] in a sample of inpatients (M = 5.48) with psychotic disorders, bipolar disorder, and MDD as well as a sample of middle-aged and older patients with schizophrenia or schizoaffective disorder (M = 5.22) [8]. The mean score, however, was much lower than that obtained in Beck et al’s [4] sample of inpatients with schizophrenia and related psychosis and MDD (with and without psychosis) (M = 7.02).

While no significant differences in SC and overall CI scores were observed across diagnostic groups in the current study, significant differences were noted for the SR subscale of cognitive insight. In particular, individuals with schizophrenia and related psychosis reported lower SR scores (i.e. lower CI) compared to those with anxiety disorders. This was in concordance with the findings from Beck et al. [4] and Bora et al. [12] whereby patients with psychosis had lower mean SR scores than patients without psychosis. These studies, however, did not compare the SR scores across other diagnostic groups as in the current study.

Other studies which have compared across diagnostic groups have primarily looked at the overall CI scores. Given that higher SC scores and lower SR scores are indicative of lower CI [5], the current findings of lower SR scores (i.e, lower cognitive insight) among those with schizophrenia and related psychosis was partly concordant with past studies such as Beck et al. [4], who found the overall CI score of inpatients with schizophrenia or psychotic depression to be significantly lower than that of patients with MDD (without psychotic features). Likewise, Colis et al. [11], found the CI scores of those with psychotic or bipolar disorder to be lower than those with MDD. Direct comparison of current findings to aforementioned studies is limited given that individuals with bipolar disorder and depression were included under the larger diagnostic group of mood disorders in the current study.

One possible reason for the lack of significant findings in SC and CI scores across diagnostic groups in this study could be due to individuals across all diagnostic groups having been under regular psychiatric treatment for a similar length of time (M = 8.46, SD = 7.45; in months). The current study population could thus, be a relatively stable and uniform population as reflected in the lack of significant difference in scores. However, it is noteworthy that the lower SR scores among individuals with schizophrenia and related psychosis mirrored past evidence which have found insight to be poorer among individuals with psychosis given the inherent cognitive difficulties that are part of the illness itself [5].

In relation to socio-demographic correlates, only age, educational level, and ethnicity were associated with CI and its subscales. Having no formal education was associated with higher SC scores (i.e., low insight), older age was associated with lower SR and overall CI scores while Malay ethnicity was associated with lower CI scores. Our study findings were partly reflective of Uchida et al’s [23] who found the SR and overall CI scores to have a significant negative association with age among Japanese patients with schizophrenia. It is possible that younger people may be more aware of mental illnesses and hence may be more open to feedback from others and might be less rigid in their beliefs as compared to older adults [24]. With regards to education, it is possible that those with no formal education may be less open to new information and more resistant to changing their beliefs (i.e., higher self-certainty) as compared to those with higher education; this result, however, requires further validation. In contrast, Bora et al. [12] found no association between CI and age, education, duration of illness or number of hospitalizations among schizophrenia patients with and without current psychotic symptoms.

With regards to ethnicity, our finding of lower CI among Malays corresponds to Qi et al. [25] who found Malays (and Indians) to have higher prejudice and misconception towards mental illness. Given the association between misconceptions and knowledge of mental illness, it is possible that this lack of knowledge may result in them being less inclined to alter their beliefs on mental illnesses. Differences in explanatory model of illness could also partly account for this finding.

While some studies have found greater insight (particularly clinical insight) to be linked to a lower QoL [3, 26], the current study found a positive correlation between CI and QoL. However, a significant finding was only found for the environment domain of QoL. Further examination of the subscales showed higher SR scores (i.e. greater insight) to be associated with higher QoL in the environment domain and higher SC scores (i.e., lower insight) to be associated with lower scores on the social relationships domain of QoL.

The environment domain is representative of areas such as physical security, financial resources, health and social care and their availability, and opportunities for acquiring new information and skills [20, 21]. It is likely that individuals with better CI (i.e., aware of their lack of objectivity or distortions and their cognitive deficiencies) will perceive more physical security and be more likely to access new information, thus exhibiting higher QoL in this domain [4, 5]. This finding was partly reflective of Phalen et al. [6] who found SR to have a positive relationship with overall QoL but in contrast with Kim et al. [10] who found higher scores on the SR subscale to be associated with lower total QoL scores and lower QoL in the psychosocial domain among the patients with schizophrenia. Caution is warranted in generalizability of findings across studies given the different measures of QoL used; while the current study assessed QoL in terms of physical health, psychological, social relationships, and environment domains, Kim et al’s [10] study used a schizophrenia-specific QoL instrument which looked at the psychosocial and vitality domains of QoL.

With respect to the SC scores, a negative association was found between SC scores and QoL in the social relationships domain (e.g., personal relationships, social support) [20, 21]. Individuals who are more resistant to feedback from others and overconfident about their beliefs of mental illness may experience less satisfying relationships and be less likely to seek out help from others due to the perceived incongruence of beliefs. This in turn, may have resulted in a lower QoL in the social relationships domain.

### Limitations

Current findings, however, should be considered in view of its limitations. Firstly, the cross-sectional nature of the study does not allow for causal inferences between CI and QoL. Secondly, individuals with bipolar disorder and depression were included under the broader diagnostic group of mood disorders. Past studies have not only shown CI to vary between individuals with bipolar disorders and psychosis but also noted differences among individuals with bipolar disorders, such that bipolar patients with a more recent episode of mania have less CI than those with bipolar disorder with a most recent episode of depression [11]. Not making this distinction might have masked some essential differences in CI across the diagnostic groups. Thirdly, conceptions of mental illness, its symptomatology and insight are likely to be strongly influenced by cultural interpretations [27]. Kao et al. [13] for instance, found the Taiwanese version of the BCIS to be similar in factor structure to the original BCIS but the items in the Taiwanese version to be distributed differently from the original scale following a principal components analysis (PCA). Hence, it is possible that cultural differences in endorsement of items may exist and this scale might have to be validated in the current context. In the current study, CI was found to differ across ethnicities; while this was partly attributed to differences in the levels of prejudice and understandings of mental illness found among the local population as reported in past studies [25], these differences were not examined in this study. Measuring the level of stigma among the different ethnicities might be important given that past studies such as Lysaker et al. [26] have found insight (clinical insight) to interact with internalized stigma to predict levels of hope, self-esteem, as well as functioning.

Despite some supporting evidence for the link between CI and QoL in this study, it is likely that this relationship is moderated by other variables. Past studies have suggested an interaction between symptoms and the level of insight to predict QoL, particularly among individuals with schizophrenia [16, 28, 29]. For instance, while Phalen et al. [6] found SR to have a direct positive association with QoL, the effect of SC on QoL was moderated by symptom severity whereby greater SC had a positive impact on QoL when the severity of symptoms was high but a negative impact on QoL when symptom severity was low. Similarly, Kim et al. [10] found that while higher levels of CI was associated with lower subjective QoL, this relationship was attenuated after controlling for symptoms. Yet other studies have suggested a positive correlation between delusional thinking and SC scores [16, 28, 30, 31]. While the lack of difference in SC and CI scores in our study population could be reflective of a relatively stable sample in terms of symptomatology, using a standardized measure to measure symptomatology and its severity would have helped to clarify if the absence of significant findings in terms of SC and CI across the diagnostic groups was due to the low levels or severity of symptoms.

In addition, studies have suggested clinical insight as a moderator between CI and QoL [4, 6, 32]. It is likely that an individual’s level of awareness of their symptoms and illness (i.e., clinical insight) will impact the extent to which one considers it necessary to observe their thought processes with regards to their illness or alter their beliefs (i.e., cognitive insight) [4, 14]. Also, individuals with high clinical insight have been shown to be more compliant with medication [29, 33, 34] which may in turn result in them being more able to manage their symptoms. This could subsequently promote greater CI (i.e., more willing to alter beliefs and receive feedback) which could then result in better functioning.

Only information regarding the primary diagnosis for the patients was collected in the current study. Given that the presence of co-morbid diagnoses is likely to have a greater detrimental effect on QoL compared to those without co-morbid diagnoses [35], not including this information on co-morbid diagnoses represents a potential limitation. Lastly, studies examining insight in relation to QoL have used both objective [16, 26] and subjective measures of QoL [10]; while observer-rated quality of life scales may be more adept in capturing functional capacity and illness severity, self-report scales are likely to incorporate affective states and subjective level distress [16]. As such, it would have been better to incorporate an objective measure of QoL in addition to the subjective QoL scale used in the current study for a better estimation of quality of life outcomes [16].

## Conclusions

Notwithstanding the aforementioned limitations, the current study was one of the few which has examined the association between cognitive insight and quality of life across various diagnostic groups. While clinical insight may be reflective of superficial beliefs and the explanations of illness received from health care providers, a deeper understanding of the cognitive deficiencies held by the individual (i.e., cognitive insight) might be necessary to produce a significant change in their underlying belief system. The amalgamation of these two forms of insight can then be used to inform therapeutic approaches to increase awareness and improve the QoL of individuals with mental illness.

## Data Availability

Data is not available for online access, however readers who wish to gain access to the data can write to the senior author Dr. Mythily Subramaniam at mythily@imh.com.sg with their requests. Access can be granted subject to the Institutional Review Board (IRB) and the research collaborative agreement guidelines. This is a requirement mandated for this research study by our IRB and funders.

## Abbreviations

BCIS: Beck Cognitive Insight Scale
CI: Cognitive Insight
IMH: Institute of Mental Health
MDD: Major Depressive Disorder
PCA: Principal Components Analysis
PSLE: Primary School Leaving Examination
QoL: Quality of Life
SC: Self-Certainty
SQoL: Subjective QoL
SR: Self-Reflectiveness
WHOQOL-BREF: World Health Organization Quality of Life-Brief Version
